# Evaluation of Fetal Kidney Measurement as an Adjunct Parameter for Gestational Age Estimation in the Second and Third Trimesters: A Cross-Sectional Study

**DOI:** 10.7759/cureus.85372

**Published:** 2025-06-04

**Authors:** Renukanandan Patil, Shailaja Bidri, Ravi Kumar Yeli, Rajasri G Yaliwal, Neelamma Patil, Shobha Shiragur

**Affiliations:** 1 Obstetrics and Gynaecology, Shri B. M. Patil Hospital, Medical College and Research Center, BLDE (Deemed to be University), Vijayapura, IND; 2 Radiology, Shri B. M. Patil Hospital, Medical College and Research Center, BLDE (Deemed to be University), Vijayapura, IND

**Keywords:** biometric parameter, fetal kidney length, gestational age, second and third trimester, ultrasonography

## Abstract

Introduction

Accurate estimation of gestational age (GA) is crucial for effective prenatal management. Conventional ultrasonographic parameters such as biparietal diameter, head circumference, abdominal circumference, and femur length lose precision in late gestation. This study evaluates fetal kidney length (FKL) as an adjunctive biometric tool for estimating GA during the second and third trimesters. The objective of this study was to assess the correlation between FKL and GA.

Materials and methods

A prospective observational study was conducted at a tertiary care center from April 2023 to April 2025, involving 300 singleton pregnancies between 20 and 40 weeks of gestation. Participants with a reliable last menstrual period (LMP) and dating scans were included. Exclusions applied to pregnancies with renal anomalies or systemic complications. FKL was measured via ultrasound in sagittal or coronal planes, and the average of both kidneys was analyzed. GA estimation based on FKL was compared with the American College of Obstetricians and Gynecologists (ACOG) reference standard and Hadlock’s formula using Pearson correlation and t-tests.

Results

Mean right and left kidney lengths were 36.27 mm and 35.26 mm, respectively, showing a strong positive correlation with GA (r = 0.844 and r = 0.805, p < 0.01). FKL measurements significantly increased with advancing gestation until 36 weeks (p < 0.0001).

Conclusion

FKL shows a strong correlation with GA and serves as a reliable adjunct in estimating gestational age in later trimesters, especially when traditional parameters are inconclusive.

## Introduction

Accurate estimation of gestational age (GA) is a cornerstone of effective prenatal care. It influences crucial decisions regarding the timing of delivery, interpretation of fetal growth, and intervention strategies for high-risk pregnancies [1]. Inaccurate GA estimation can result in iatrogenic preterm birth or postmaturity, leading to increased perinatal morbidity and mortality. Traditionally, GA assessment relied on the first day of the last menstrual period (LMP), uterine size, and physical examination techniques like McDonald’s rule or radiographic evaluation of ossification centers. However, these approaches are often imprecise due to individual variation in menstrual cycles and limited applicability in later pregnancy [2]. Consequently, obstetric ultrasonography has become the gold standard for determining GA.

Understanding GA is particularly vital for planning invasive procedures like amniocentesis or chorionic villus sampling, scheduling timely deliveries, and managing fetal anomalies [2]. In high-risk pregnancies, such as those with severe preeclampsia, intrauterine growth restriction (IUGR), placenta previa, or Rh incompatibility, accurate GA is critical to prevent unnecessary fetal compromise or delay in intervention [1]. Yet, traditional biometric parameters such as crown-rump length, biparietal diameter, head circumference, abdominal circumference, and femur length gradually lose accuracy as pregnancy advances, particularly in the third trimester [3,4]. This variability presents a clinical challenge in late gestation, especially when LMP is unreliable due to factors like menstrual irregularities, lactational amenorrhea, early pregnancy bleeding, or contraceptive failure [3,4].

Ultrasound has revolutionized obstetric care with its non-invasive, cost-effective, and radiation-free diagnostic capability. It plays a crucial role in assessing fetal well-being, aiding early detection of anomalies, and improving maternal and fetal outcomes, especially in resource-limited settings such as India [5,6]. Over the last two decades, alternative sonographic markers have been explored for GA estimation, including transverse cerebellar diameter, fetal foot length, ossification centers, amniotic fluid volume, and placental grading [7]. Among these, the measurement of fetal kidney length (FKL) has emerged as a promising tool. MRI studies and early ultrasonographic evaluations have shown a linear increase in FKL with GA, suggesting its potential as an adjunct GA estimator [8].

FKL is easily identifiable, simple to measure, and, importantly, maintains a consistent and linear growth pattern during the second and third trimesters. Studies have reported a sensitivity of approximately 86% and a specificity of about 90% for FKL in accurately estimating GA. Compared to traditional biometric parameters that fetal growth abnormalities may influence, FKL offers a more stable indicator of gestational progression, particularly after the 24th week [9]. It has been found to correlate well with GA, sometimes outperforming conventional parameters after the 24th week of gestation [10,11]. This correlation opens new possibilities in cases where standard parameters provide conflicting or inconclusive data. Originally studied to assess renal morphology and detect anomalies, recent research has increasingly focused on its role in estimating GA [12,13].

The present study was conducted to evaluate the effectiveness of FKL as an additional biometric parameter for estimating GA, particularly in the second and third trimesters. By comparing its accuracy with established sonographic markers such as biparietal diameter, head circumference, abdominal circumference, and femur length, this study aims to validate the role of FKL as a reliable, supplementary tool for improving GA estimation. Its adoption may enhance clinical decision-making, especially in pregnancies with uncertain dates or atypical fetal growth patterns.

## Materials and methods

This cross-sectional observational study was conducted in the Department of Obstetrics and Gynaecology at BLDE (Deemed to be University), Shri B. M. Patil Medical College and Research Centre, Vijayapura. The study was carried out over a period of two years, from April 1, 2023, to April 1, 2025. Ethical clearance for the study was obtained from the Institutional Ethics Committee, BLDE (Deemed to be University) (approval number: BLDE(DU)/IEC/912/2023-24). Eligible participants were enrolled after obtaining informed consent.

Eligibility criteria

The study population comprised pregnant women attending the outpatient department for routine antenatal care, with GA between 20 and 40 weeks. A total of 300 participants were enrolled based on the eligibility criteria. Inclusion criteria were singleton pregnancies between 20 to 40 weeks of gestation with a known last menstrual period (LMP) and a dating scan performed appropriately. Exclusion criteria included pregnancies with obstetric or medical complications such as hypertensive disorders of pregnancy, gestational diabetes, IUGR, cardiac disease, or epilepsy, pregnancies with inherited or congenital renal anomalies, including polycystic kidney disease, multicystic dysplastic kidney, and ectopic kidneys, and those without a reliable LMP or dating scan. Cases of pyelectasis exceeding 10 mm were also excluded. 

Sample size calculation

Assuming a confidence interval of 95%, a 5% level of significance, and a margin of error of 0.5, the sample size was calculated to be 300, using the formula: $n = \left( \frac{Z \times \sigma}{d} \right)^2$, where Z = 1.96 (z-score for 95% CI), σ = 4.2 (standard deviation), and d = 0.5 (margin of error).

Data collection

GA at enrolment was determined using the American College of Obstetricians and Gynecologists (ACOG) application, which integrates both the known LMP and data from a correctly timed dating scan; this served as the standard GA reference. Each participant then underwent a routine growth scan, during which the fetal GA was estimated using Hadlock's formula, based on biometric parameters. The lengths of both fetal kidneys were measured individually in mm in either the sagittal or coronal plane (supero-inferior dimension) using a high-resolution ultrasound machine (Voluson E8, 3.5-5 MHz curvilinear transducer; GE Healthcare Technologies, Inc., Chicago, Illinois, United States). To minimize inter- and intra-observer variability, all measurements were performed by a single experienced radiologist, and each measurement was taken three times with the average recorded for analysis. The mean of both renal lengths was calculated, and GA was estimated based on the hypothesis that FKL in mm corresponds to GA in weeks.

**Figure 1:**
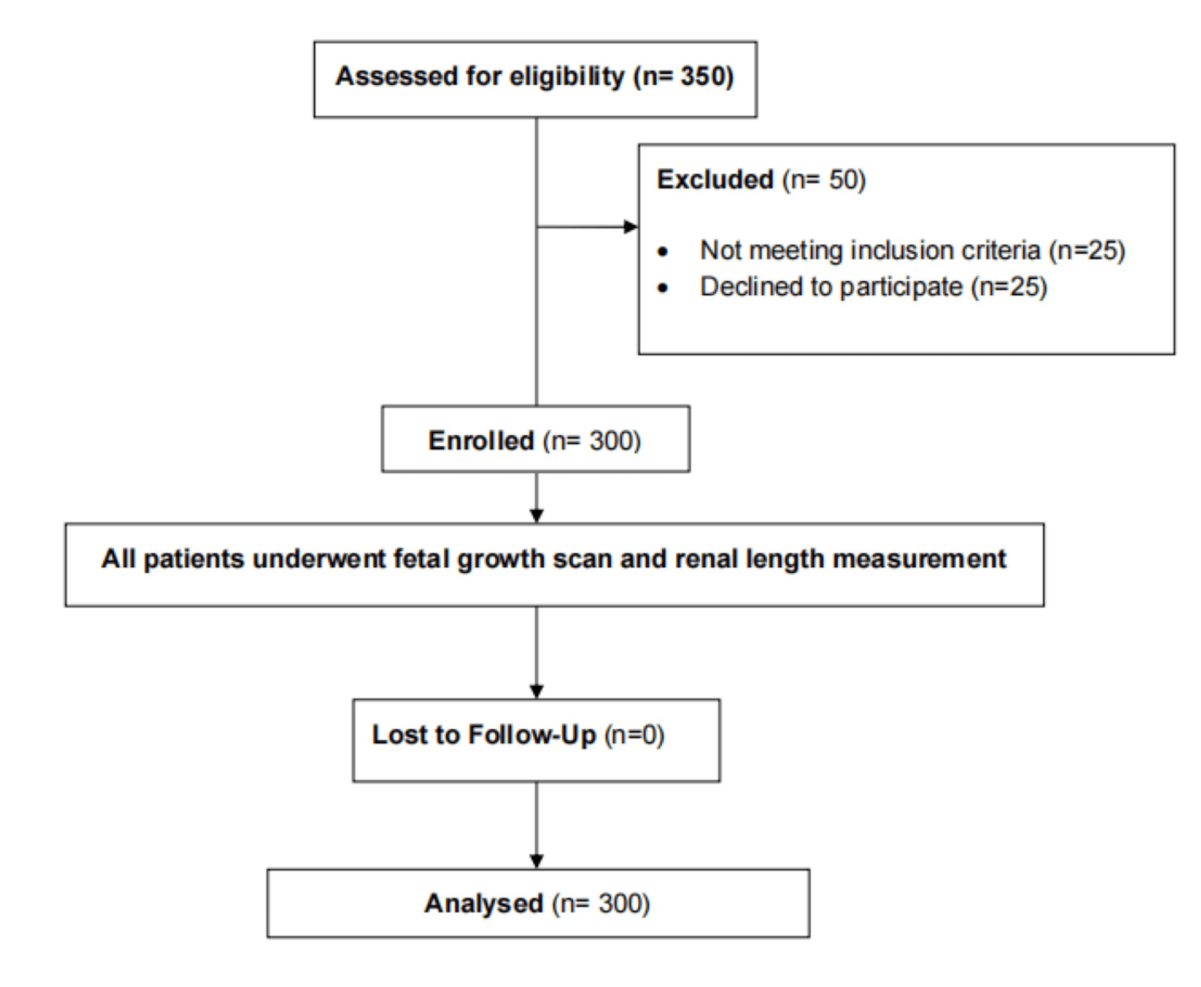
Study flowchart

The study process is given in Figure 1.

Data analysis

The GAs derived from renal length measurements and Hadlock’s formula were compared with the standard GA obtained via the ACOG method. Data were recorded in Microsoft Excel (Microsoft Corporation, Redmond, Washington, United States) and statistically analyzed using IBM SPSS Statistics for Windows, Version 26 (Released 2019; IBM Corp., Armonk, New York, United States). Categorical variables were expressed as frequencies and percentages. Continuous variables were expressed as mean and standard deviation (SD) and compared using t-tests. Pearson correlation analysis was performed to assess the strength of the association between GA and FKL. A p-value of <0.05 was considered statistically significant.

## Results

A total of 300 antenatal women between 20 and 40 weeks of gestation were enrolled in the study. Each participant underwent a single ultrasound evaluation; no follow-up scans were performed. The cross-sectional analysis of this dataset allowed for evaluation of the relationship between FKL and GA across a wide range of gestational weeks. The majority of participants were aged 21-25 years, and more than half were multiparous. Most women presented with a fundal height corresponding to term gestation (Table 1).

**Table 1:** Baseline characteristics of study participants (N = 300) Categorical variables were expressed as frequency and percentages.

Characteristic	Category	Frequency	Percentage
Age (years)	<20	42	14.0%
	21–25	155	51.7%
	26–30	76	25.3%
	>30	27	9.0%
Obstetric Score	Primi	125	41.7%
	Multipara	175	58.3%
Fundal Height	28–30 weeks	7	3.0%
	32–34 weeks	26	8.7%
	34–36 weeks	110	36.7%
	Term size	157	52.35

The mean height, weight, and abdominal circumference were within expected antenatal norms. (Table 2)

**Table 2:** Anthropometric measures Continous variables were expressed as mean and SD.

Anthropometric Measures	Mean ± SD
Height (cm)	154.83 ± 4.07
Weight (kg)	58.00 ± 4.17
Abdominal Circumference (cm)	87.90 ± 3.50

Fetal kidney measurements showed consistent and symmetrical growth patterns, with the mean right kidney length slightly higher than the left. The average of both lengths was used as the mean renal length for analysis (Table 3).

**Table 3:** Distribution of kidney length among study participants Continuous variables were expressed as mean and SD.

Variable	Mean (mm)	Standard Deviation (mm)
Right Kidney Length	36.27	2.20
Left Kidney Length	35.26	2.37
Mean Kidney Length	35.76	2.24

A statistically significant increase in FKL was noted with advancing GA, particularly between 28 and 36 weeks. However, the increase observed at 38 weeks was not statistically significant (Table 4).

**Table 4:** Association between gestational age and fetal kidney length Continuous variables were expressed as mean and standard deviation and compared using t-tests. A p-value less than 0.05 was considered statistically significant.

Gestational Week	Right Kidney Length (Mean ± SD)	Left Kidney Length (Mean ± SD)	t-value	P-value
28 weeks	29.00 ± 1.15	28.50 ± 2.38	4.19	<0.0001
32 weeks	32.06 ± 1.14	30.88 ± 1.40	5.11	<0.0001
34 weeks	34.88 ± 1.05	33.70 ± 1.40	6.17	<0.0001
36 weeks	36.24 ± 0.94	35.40 ± 1.10	5.58	<0.0001
38 weeks	38.02 ± 1.30	37.00 ± 1.40	1.29	0.2006

Correlation analysis demonstrated a strong positive relationship between gestational age and fetal kidney length, confirming renal length as a potential marker for estimating gestational age (Table 5).

**Table 5:** Correlation between gestational age and kidney length Pearson correlation analysis was performed to assess the strength of the association between gestational age and kidney lengths. A p-value of <0.05 was considered statistically significant.

Variables Correlated	Pearson Correlation Coefficient (r)	P-value
Gestational Age vs. Right Kidney	0.844	<0.01
Gestational Age vs. Left Kidney	0.805	<0.01
Gestational Age vs. Mean Kidney	0.840	<0.01

## Discussion

This study evaluated the accuracy of FKL measured via ultrasound in the second and third trimesters as a means of estimating GA. Our results indicate a strong positive correlation between GA and FKL, reinforcing previous research that supports the use of FKL as a reliable parameter in obstetric ultrasonography.

The majority of participants in our study were aged 21-25 years (51.7%) and were multigravida (58.3%), consistent with population trends in antenatal cohorts. A substantial portion of participants were at term (52.3%), followed by 34-36 weeks (36.7%), reflecting a good distribution across late GAs. While we observed some alignment with previous studies in terms of participant characteristics, direct comparison was limited by differences in reporting formats. For example, Deeluea et al. described a progressive increase in fundal height from 19.1 cm at 20 weeks to 35.4 cm at 40 weeks, but did not stratify participants by fundal height bands, limiting comparability with our fundal height subgroup analysis [14].

Our study reported a mean right kidney length of 36.27 mm (SD = 2.20), left kidney length of 35.26 mm (SD = 2.37), and a mean combined FKL of 35.76 mm (SD = 2.24). These values are in line with findings from Gautam et al., who reported a mean FKL of 36.8 mm (SD = 4.02) at a mean GA of 35.5 weeks, suggesting consistency in FKL growth across gestation [15].

Moreover, our results showed a statistically significant increase in both right and left kidney lengths from 28 to 36 weeks (p < 0.0001), with no significant difference noted at 38 weeks (p = 0.2006). This trend mirrors the findings by Kiridi et al., who also documented consistently greater right kidney measurements and observed significant inter-kidney differences at earlier gestational stages [16]. Additionally, our observation of non-significant kidney length differences at 38 weeks is consistent with the findings reported by Purohit et al., who similarly noted a plateau in renal growth and a lack of significant inter-kidney variation at term gestation [17].

Further reinforcing our conclusions, Edevbie and Akhigbe reported that FKL increased steadily from 20.87 ± 0.75 mm at 20 weeks to 41.41 ± 0.07 mm at 41 weeks, with a strong correlation (r = 0.997, p = 0.000) between FKL and GA [18]. Similarly, Gautam et al. noted a strong correlation (r = 0.921, p = 0.001) and improved GA prediction when combining FKL with conventional parameters [15]. Our data aligns with these findings, as we observed a correlation coefficient of r = 0.844 between right kidney length and GA (p < 0.01), and r = 0.89 (p = 0.001) for mean kidney length, confirming the robustness of FKL as a GA marker.

Limitations

This study is not without limitations. Firstly, the potential influence of maternal comorbidities (such as gestational diabetes or hypertensive disorders) on fetal kidney development was not accounted for, which could have affected the measurements. Secondly, the data were collected from a single tertiary care center, limiting generalizability. Thirdly, our analysis excluded first-trimester data, which could have provided a more complete picture of early renal growth and its correlation with GA. Additionally, this study did not include a direct comparison of FKL with conventional biometric parameters such as biparietal diameter, femur length, and abdominal circumference, which is necessary to establish FKL as a reliable adjunct parameter for GA estimation. Future research should address this gap to better validate the clinical utility of FKL.

## Conclusions

This study demonstrates that FKL is a valuable adjunct parameter for estimating GA during the second and third trimesters. Its strong correlation with GA highlights its potential utility, particularly in situations where conventional biometric markers may be unreliable or inconclusive. The findings contribute to the growing body of evidence supporting FKL as a reliable and reproducible tool in prenatal evaluation. However, further studies involving larger and more diverse populations are warranted to validate these results and establish standardized clinical guidelines for their routine use in obstetric practice.
